# Antibacterial and anatomical defenses in an oil contaminated, vulnerable seaduck

**DOI:** 10.1002/ece3.7996

**Published:** 2021-08-19

**Authors:** Anders Pape Møller, Karsten Laursen, Jorge Izaguirre, Alfonso Marzal

**Affiliations:** ^1^ Ministry of Education Key Laboratory for Biodiversity Science and Ecological Engineering College of Life Sciences Beijing Normal University Beijing China; ^2^ Ecologie Systématique Evolution CNRS Université Paris‐Saclay Orsay Cedex France; ^3^ Department of Bioscience Aarhus University Ronde Denmark; ^4^ Department of Zoology University of Extremadura Badajoz Spain

**Keywords:** diving, ducks, ingestion, intestine, pollution, selection

## Abstract

Oil spills have killed thousands of birds during the last 100 years, but nonlethal effects of oil spills on birds remain poorly studied. We measured phenotype characters in 819 eiders *Somateria mollissima* (279 whole birds and 540 wings) of which 13.6% were oiled. We tested the hypotheses that (a) the morphology of eiders does not change due to oil contamination; (b) the anatomy of organs reflects the physiological reaction to contamination, for example, increase in metabolic demand, increase in food intake, and counteracting toxic effects of oil; (c) large locomotion apparatus that facilitates locomotion increases the risk of getting oiled; and (d) individual eiders with a higher production of secretions from the uropygial grand were more likely to have oil on their plumage. We tested whether 19 characters differed between oiled and nonoiled individuals, showing a consistent pattern. The final model retained seven predictor variables showing relationships between eiders contaminated with oil and food consumption, flight, and diving abilities. We tested whether these effects were due to differences in body condition, liver mass, empty gizzard mass, or other characters that could have been affected by impaired flight and diving ability. There was no evidence of such negative impact of oiling on eiders. We found that significant exposure to oil was associated with increased diversity of antibacterial defense. Oiled eiders did not constitute a random sample, and superior diving ability as reflected by large foot area was at a selective disadvantage during oil spills. Thus, specific characteristics predispose eiders to oiling, with an adaptation to swimming, diving, and flying being traded against the costs of oiling. In contrast, individuals with a high degree of physiological plasticity may experience an advantage because their uropygial secretions counteract the effects of oil contamination.

## Graphical abstract

Oiled eiders did not constitute a random sample and superior diving ability was at a selective disadvantage during oil spills. Individuals with a high degree of physiological plasticity may experience an advantage because their uropygial secretions counteract the effects of oil contamination.

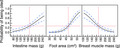

## 1 INTRODUCTION

Human activity leads to pollution and reductions in the viability of wild animals. Of these activities, oil pollution has caused the death of thousands of birds. Analyses of the consequences of oil pollution have shown significant reductions in juvenile and adult survival rates in areas where breeding, molting, and wintering take place (Burger, 2018; Votier, Birkhead, et al., 2005; Votier, Hatchwell, et al., 2005). Open oil wells containing extra‐heavy crude oils occur naturally, and birds are known to approach such sites for resting and foraging (Burger, 2018). Hence, it is possible that some birds may have evolved adaptive responses to naturally occurring oil. Analyses of the effects of oil spills on birds are generally based on the assumption that individual birds affected by pollutants constitute a random sample of the overall population (Joensen, 1972). Contamination with oil often damages the plumage and eventually to reduced condition that may result in death (Camphuysen, 2010; Dunnet et al., 1982; Paine et al., 1996; Ramseur, 2017; Teal & Howarth, 1984). Ingestion of oil via food items can also cause physiological damages and death in seabirds (Dean & Bursian, 2017; Horak et al., 2017). While hundreds of thousands of birds have been affected by oil spills (Dunnet et al., 1982; Teal & Howarth, 1984), there are still gaps in our knowledge of factors associated with the consequences of oil pollution for birds and other organisms.

Oil spills have been a significant cause of mortality in seabirds, but also affect other organisms (Dunnet et al., 1982; Teal & Howarth, 1984; Paine et al., 1996; Widdows et al., 2002; Bautista & Rahman, 2016; Ramseur, 2017). The Danish waters are among the busiest shipping lanes in Europe connecting the North Sea and the Baltic Sea, and large oil spills have taken place with more than 50,000 sea ducks killed during 1968–1971 mostly being eiders *Somateria mollissima* (Joensen, 1972). More recently, in 2008 and 2010 more than hundred oil spills were confirmed in Danish waters, with spill volumes up to 1,000 m^3^ (Schulz et al., 2017). However, the rate of oiling in seabirds has generally decreased in the Danish waters as well as in the southern North Sea in recent decades (Schulz et al., 2017; Waltho & Coulson, 2015). Oil spills are known to have ecological effects on a diverse range of organisms (Teal & Howarth, 1984), sometimes resulting in long‐term effects on ecosystems (Peterson et al., 2003). In contrast, there is little information on differential effects of oil pollution on individuals varying in age, sex, or phenotype. In particular, demographic variables such as the proportion of nonbreeders and adult survival rates have been suggested to be severely affected by oil spills (Votier, Birkhead, et al., 2005; Votier, Hatchwell, et al., 2005; Wolfaardt et al., 2009). Finally, while several studies have focused on birds killed by oil pollution, fewer studies have examined the physiological and plumage consequences for individuals that survived oil spills (Alonso‐Alvarez et al., 2007; Bianchini & Morrissey, 2018; Champoux et al., 2020; Horak et al., 2017, 2020).

The secretions of the uropygial gland are used by birds for preening the plumage and for maintenance of feathers (Møller & Laursen, 2019a). Comparative analyses have shown positive relationships between the size of the uropygial gland and risk of predation and risk of hatching failure (Møller et al., 2010, 2019). For example, it has been shown that barn swallows with larger uropygial glands reared a greater total number of fledglings when living in environments with higher abundance of conspecifics (Møller et al., 2010). Eiders may preen their plumage with secretions from the uropygial gland which contain antibacterial material that may help cleaning feathers in general and especially feathers contaminated by oil.

We focused on the eider belonging to the Baltic/Wadden Sea population that has declined during recent decades (Ekroos et al., 2012). The objectives of this study were to analyze the effects on eiders that were contaminated by oil but have survived. We did so by testing for differences in morphology between eiders that were oiled and those belonging to a nonoiled control group. The morphology of bird species has a specific size throughout their lifetime, in contrast to the size of organs that can change within weeks due to season and environmental conditions (Laursen et al., 2019; Piersma et al., 1996). Thus, we assume that the morphology of eiders reflects the condition of individuals when they become contaminated by an oil spill and remain stable afterward, which may be opposite to the anatomy of organs that reflects the physiological reaction following contamination to increase or stabilize metabolic function and counteract the toxic effects of oil on feathers. From this, we hypothesize that (a) the morphology of eiders does not change due to oil contamination; (b) the anatomy of organs reflects the physiological reaction to contamination, for example, increase in metabolic demand, increase in food intake, and counteracting toxic effects of oil; (c) individuals with a large locomotion apparatus have an increased risk of getting oiled compared to individuals with a small locomotion apparatus; and (d) individual eiders with a higher production of secretions from the uropygial gland will likely have oil on their plumage. These analyses were based on eiders collected at the same sites where some eiders were not contaminated with oil and some were contaminated recently or at least contaminated during the same winter. All individual eiders had survived so far and had been able to fly before they were shot for sampling. Sampling of eiders in the study was part of a study of the effects of other environmental conditions than oil spills on eiders, and thus, eiders with or without oil on their feathers were not the focus and thus supposed to be sampled randomly.

## 2 METHODS

### 2.1 Data sets

**FIGURE 1:**
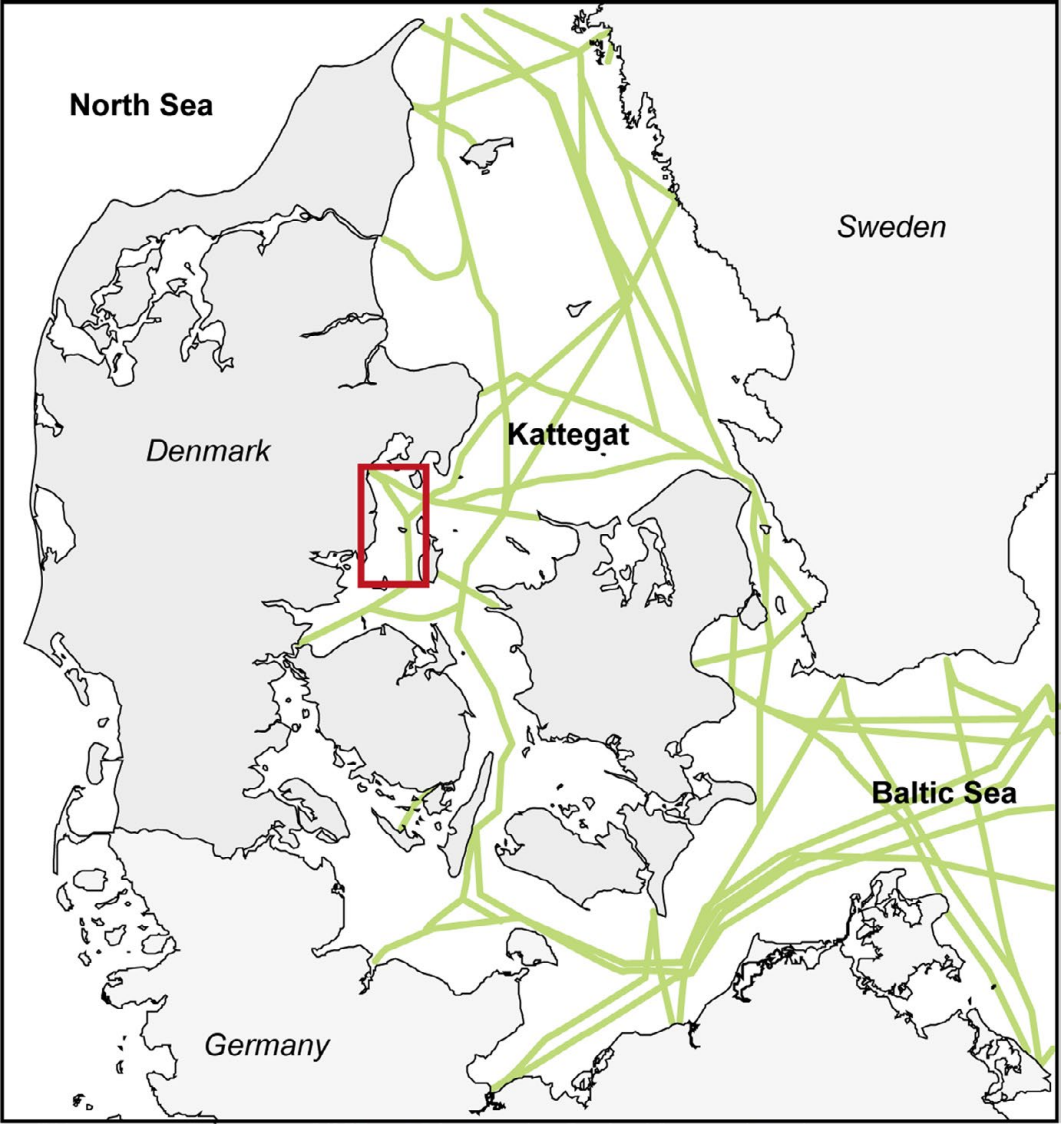
Map showing the main study area (red square) and the main ship traffic lanes through the Danish waters between the North Sea and the Baltic Sea (Helcom, 2018)

We obtained samples of shot eiders from Denmark during 2014–2019. Eiders outside the hunting season were collected under license from the Danish Ministry of the Environment (SN‐302‐009, SNS‐3446‐00103, NST‐3446‐00018, NST‐3465‐00007). The eiders were sampled during 1 October–10 March by hunters at various localities in Denmark with most birds from the eastern part of Kattegat (Figure 1). We had a sample of 819 eiders of which 279 were whole specimens and 540 were wings sent in by hunters to the Danish Wing Survey (Christensen et al., 2017). All eiders were frozen the same day they were shot or early next day. The wings were frozen at the day they were received. Later in the laboratory, bodies and wing feathers were inspected visually for oil contamination as reflected by the feathers being sticky with a wet and shiny appearance rather than smooth and dry. Visual inspection of the plumage has been used to assess whether birds are contaminated with oil (Joensen, 1972; Paruk et al., 2019). The method has been recommended or used by the Trilateral Monitoring and Assessment Program for the Wadden Sea, by OSPAR, the Oslo‐Paris Commission, by US Wildlife Service and in Greenland in monitoring programs of oiled seabirds washed ashore (Berg, 2005; Camphuysen, 2005; Schulz et al., 2017; Wegeberg et al., 2017).

Oil contamination of seabirds occurred following cleaning of tanks on ships despite such contamination being illegal. A major pollution event by a container ship occurred on 10 February 2017 in southern Kattegat, while other oil spills were of unknown origin, and not detected by the national survey.

Of 817 eiders inspected for oil, 111 specimens (13.6%) had oil on their plumage. All eiders were sexed and aged using standard plumage characteristics (Cramp et al., 1982). The age of female eiders can be identified up to the third calendar year (given the value 1–3) and male eiders to the sixth calendar year (given the values 1–6).

Worn feather tips and fast growing feathers indicate individuals with weak feathers (Møller et al., 2018). Thus, for entire eiders and wings, we removed the second primary and recorded its length with a ruler to the nearest mm and the width of the feather shaft (rachis) with a digital caliper to the nearest 0.01 mm. We determined whether the tip of the second primary was broken and had an uneven edge resulting in a clearly worn feather.

We measured daily growth increments on the second primary by placing it on a piece of white paper on top of a flat surface of styro‐foam under dim light (Grubb, 2006). We marked the place where the first dark and light band started with an insect pin and then counted five such bands before making a second mark with the pin. We measured the distance between these two marks with a digital caliper to the nearest 0.01 mm and then divided this measure by five to obtain an estimate of the average width of a single daily growth increment (see Møller & Laursen, 2019b).

We obtained estimates of aspect ratios for eiders as [(wing span)^2^/(wing area)]. Wingspan of the fully stretched wing was measured with a ruler to the nearest mm according to Pennycuick’s (2008) recommendations. Wing area was calculated by first accurately drawing the profile of one wing on a piece of paper with the leading edge of the wing being held at an angle of 90º to the axis of the body and subsequently cutting the profile, weighing it on a precision balance and converting the weight to wing area using the weight of a reference surface of known area. Our estimates of wing area included the so‐called root box that is the area of the body between the wings (see Pennycuick, 2008 for details).

Entire eiders were weighed to the nearest 0.1 kg. The uropygial gland and the spleen from each specimen were removed. These glands were immediately weighed on a precision balance to the nearest 0.001 g. Likewise, we weighed heart, liver, breast muscle, intestine, empty gizzard, gizzard content to the nearest g, the length, width, and depth of the head to the nearest 0.01 mm, the length, width, and depth of the beak to the nearest 0.01 mm, and the longest testes and yolk with a digital caliper to the nearest 0.01 mm. Head volume, which is an index of brain mass (Jaatinen et al., 2019), was calculated as the product of head length, width, and height, multiplied by π and 4/3 (the latter is the constant used for estimating the volume of an ellipsoid). Likewise, beak volume was estimated as length, width, and depth of the beak to the nearest 0.01 mm, multiplied by π and 4/3 (the latter is the constant used for estimating the volume of an ellipsoid). For statistics for all characters, see ESM. Morphological and anatomical measurements were recorded in the laboratory by APM and KL.

### 2.2 Estimation antimicrobial activity from the uropygial gland

We removed the uropygial grands from 119 eiders and extracted uropygial secretions to analyze antibacterial activity. First, we defrosted the gland for 10 min. Because it was impossible to extract secretions without destroying the gland, we made an incision in the middle of the gland and scraped the gland in order to obtain a small quantity of solution. Subsequently, we refroze the gland and stored the solution at −20°C until flow cytometry analyses.

The uropygial secretion was extracted and added to 1 or 2 μl to each well in a 96‐well plate. A total of 200 μl per well of bacterial suspension (*Staphylococcus epidermidis*) (ATCC^®^ CRM–12228™) were then dispensed in the 96‐well plate. The determination of antimicrobial effects by flow cytometry in bacteria usually requires 24–48 hr of culture incubation for bacterial growth at 37°C (Lukomska‐Szymanska et al., 2017; Martins‐Oliveira et al., 2020; O'Brien‐Simpson et al., 2016). After culture incubation for bacterial growth at 37°C for 24 and 48 h, we used flow cytometry for detecting absolute cell count and hence assessed antimicrobial activity of the uropygial gland secretion against *S. epidermidis*. This technique is a rapid, accurate, and highly reproducible methodology widely used to estimate antimicrobial activity (Alvarez‐Barrientos et al., 2000; Magallanes et al., 2016; Marzal et al., 2018). To summarize, we recorded four measurements of antibacterial activity (Aba) from each individual eider (a) Aba of 1 μl secretion for 24 hr; (b) Aba of 1 μl secretion in 48 hr; (c) Aba of 2 μl secretion in 24 hr; and (d) Aba of 2 μl secretion in 48 hr. The concentration of secretion and time of incubating were repeated for double doses and time as a control for the incubation process. All analyses of uropygial secrets and antibacterial response were made by AM and JI.

### 2.3 Statistical analyses

We developed general linear models (GLM) including main effects (Tables 1 and 2). These main variables with the smallest variables were eliminated until all predictors included had associated *p*‐values < .05 using a backward elimination procedure. We used GLM's to predict the probability of eiders being oiled assuming that the data were binomially distributed with an identity link function (SAS, 2012). We evaluated the strength of relationships using Pearson's correlation coefficients (Cohen, 1988; Rosenberg, 2010). We tested whether the incidence of getting oiled differed among age classes using Levene's test.

**TABLE 1:** Generalized linear models with a binomial distribution and a logit link function of oil pollution in eiders for 19 different phenotypic characters grouped into general characters, morphological characters, and anatomical characters and others

Character	LR χ^2^	Estimate	*SE*	Lower CL	Upper CL	*p*	Goodness of fit	*df*
General characters								
Sex	13.94	0.484	0.122	0.240	0.719	.0002	813.55	815
Age	6.41					.268	803.69	811
Date (1 = 1 October)	305.46	0.142	0.020	0.106	0.186	<.0001	248.39	715
Morphological characters								
Feather mass (g)	12.78	5.103	1.443	2.295	7.959	.0003	840.87	808
Shaft thickness (mm)	12.82	−0.112	0.030	−0.170	−0.052	.0003	803.94	815
Feather growth band (mm)	5.51	−0.543	0.237	−1.017	−0.088	.019	815.09	813
Foot area (mm^2^)	35.86	0.193	0.037	0.123	0.270	<.0001	268.87	274
Gizzard mass (g)	5.19	0.009	0.004	0.001	0.016	.112	278.97	277
Aspect ratio	2.53	0.189	0.121	−0.043	0.432	.016	273.40	270
Body mass (kg)	29.16	3.437	0.688	2.133	4.838	<.0001	279.34	275
Wing length (mm)	9.76	−0.032	0.010	−0.052	−0.012	.0018	817.31	814
Wing loading	22.73	153.78	34.41	88.43	223.73	<.0001	274.39	242
Wear index	37.26	1.013	0.020	0.649	1.413	<.0001	804.02	815
Anatomical characters								
Breast muscle (g)	1.81	−0.009	0.007	−0.022	0.004	.179	277.41	275
Liver mass (g)	4.86	0.018	0.008	0.002	0.035	.028	275.73	274
Gizzard content (g)	23.20	0.021	0.005	0.012	0.032	<.0001	277.69	272
Spleen mass (g)	9.27	−0.766	0.263	−1.299	−0.266	.0023	269.26	269
Intestine mass (g)	17.77	0.016	0.004	0.009	0.025	<.0001	277.28	275
Uropygial gland mass (g)	12.01	0.501	0.150	0.214	0.803	.0005	221.37	223

Note

Likelihood ratio (LR) chi‐square, estimates, *SE*, and 95% confidence limits (CL), and probabilities are also reported. The goodness of fit is given as likelihood ratio chi‐square and the associated *df*.

**TABLE 2:** Generalized linear model with a binomial distribution and a logit link function of probability of oil pollution in relation to beak volume, foot area, wing length, breast muscle, heart mass, gizzard content, and intestine mass

Character	LR χ^2^	Estimate	*SE*	Lower CL	Upper CL	*p*
Beak volume (mm^2^)	7.71	0.485	1.728	1.456	8.350	.005
Foot area (cm^2^)	39.02	0.268	0.051	0.173	0.375	<.0001
Wing length (mm)	7.95	−0.038	0.014	−0.066	−0.011	.048
Breast muscle mass (g)	23.44	−0.047	0.010	−0.069	−0.027	<.0001
Heart mass (g)	5.60	0.139	0.059	0.018	0.024	.018
Gizzard content (g)	12.21	0.018	0.006	0.007	0.031	.0005
Intestine mass (g)	8.82	0.015	0.005	0.005	0.025	.003

Note

Estimates, *SE*, and 95% confidence limits (CL) are also reported. The model had the likelihood ratio (LR) χ^2^ = 98.36, *df* = 7, *p* < .0001. Goodness of fit was LR χ^2^ = 306.02, *df* = 261.

We estimated repeatability of length of daily growth increments of feather bands by using multiple measurements for the same individual (Falconer & Mackay, 1996). We used the correlation matrix to perform a principal component analysis on the measurements of antibacterial activity. The PC had two axes with eigenvalues of 1.81 and 1.65, respectively, accounting for an accumulated percentage of variance explaining 44.53% and 41.94%, respectively. The first axis accounted for 0.84 of the variance for 48 hr and 1 μl and 48 hr and 2 μl and loadings of 0.82 for 24 hr and 1 μl and 0.78 for 24 hr and 2 μl. We used the values for PC1 and PC2 in the following analyses.

We estimated correlations among variables to avoid problems of multicollinearity. However, variance inflation factors were all <3 implying that there were no problems of collinearity (McClave & Sincich, 2003). All analyses were made using JPM (SAS, 2012).

## 3 RESULTS

The probability of getting oiled was larger in females at 25.9% (95% CI 18.6% to 34.7%) than in males at 11.7% (95% CI 9.5% to 14.4%; Table 1), but the probability of getting oiled did not differ in older eiders compared to yearlings (Table 1; LR = 6.41, *df* = 5 and *p* = .268). The variance in probability of getting oiled decreased with age (Levene's test, *F* = 7.59, *df* = 5, 467, *p* < .0001). Finally, the probability of getting oiled increased strongly with date since 1 October, when large number of eiders occurs at the winter grounds in Denmark (Table 2).

There was no significant variation among wells in antimicrobial activity following 24 hr of incubation (LR χ^2^ = 79.70, *df* = 95, *p* = .87) and 48 hr of incubation (LR χ^2^ = 115.04, *df* = 95, *p* = .08). There were significant effects among plates after 24‐hr incubation (LR χ^2^ = 48.14, *df* = 3, *p* < .0001) and after 48‐hr incubation (LR χ^2^ = 47.29, *df* = 3, *p* < .0001).

We tested 19 morphological and anatomical variables for relationships with the probability of being oiled (Table 1). For morphological characters, we found that eiders with heavy feathers, large foot area, large gizzard mass, large aspect ratio, large body mass, large wing loading, and large wear index had a higher probability of getting oiled (Table 1; Møller & Laursen, 2019b). Eiders with thick shafts, rapid feather growth, and long wings were less likely to get oiled (Table 1).

For the anatomical variables, we found that the probability of eiders reacting to oil increased with the relative mass of the liver, the content (amount of food) in the gizzard, and the mass of the intestine implying high rates of food consumption (Table 1). The probability of reacting to oil increased with the mass of the liver (Table 1). In addition, the probability of reacting to oil decreased with the mass of breast muscles (Table 1). The mass of the spleen decreased with the probability of reacting to oil (Table 1). Further, the probability of reacting to oil increased with the mass of the uropygial gland (Table 1). Finally, we only found a marginal effect of probability of oiling on overall body condition (likelihood ratio (LR) χ^2^ = 5.01, *df* = 1, *p* = .025; effect size Pearson *r* = .228), suggesting a weak effect.

**FIGURE 2:**
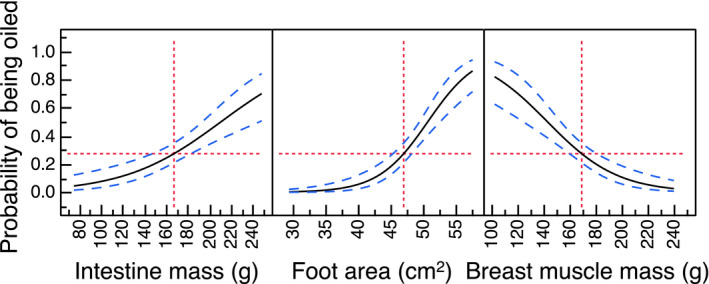
Probability of eiders having oiled feathers in relation to intestine mass (g), foot area (cm^2^), and breast muscle mass (g). The black line shows the estimated relationship, the red lines are mean values, and the blue lines 95% confidence intervals

The best fit model that accounted for the largest amount of variance included seven characters (Table 2). We evaluated the strength of the relationships using Pearson's correlation coefficients (Cohen, 1988; Rosenberg, 2010). The probability of getting oiled increased with the size of the intestine (Table 1; Figure 2). Foot area had an intermediate effect on the risk of being oiled accounting for 15% of the variance (Table 2, Figure 2). Furthermore, the probability of getting oiled decreased with breast muscle mass, accounting for 9% of the variance (Table 2, Figure 2). There were intermediate effect sizes for beak volume, wing length, heart mass, and gizzard content accounting for 2% to 5% of the variance (Table 2).

**FIGURE 3:**
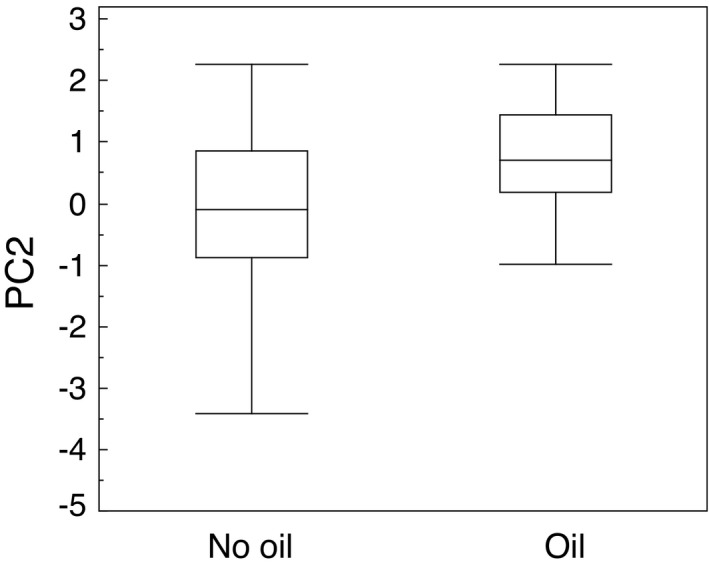
Diversity of antibacterial defense (PC2) in relation to oil pollution of eiders. The box plots show median, quartiles, 5 and 95 percentiles, and extreme values

There was a positive relationship between PC2 (variance in antibacterial activity) and whether an eider was contaminated with oil, which implies a greater variance in amount of antimicrobial defense (Figure 3; LR χ^2^ = 11.23, *df* = 1, *N* = 169, *p* = .0008, estimate (SE) = 0.677 (0.225)), while there were no additional effects of sex, age, body mass, femur length, and locality. This implies that oil contamination was associated with higher release of secretions from the uropygial gland.

## 4 DISCUSSION

We found that seven out of 19 characters differed between oiled and nonoiled individuals, showing a consistent pattern. The seven predictor variables were all linked to food consumption, flight, and diving abilities, hence having a clear functional role in the daily life of eiders. Finally, we showed whether these effects were due to differences in body condition, liver mass, empty gizzard mass, or other characters that could have been affected by impaired flight and diving ability.

We found no evidence for the hypothesis that antimicrobial defense is positively correlated with oil pollution. However, we did find that significant exposure to oil was associated with increased diversity of antibacterial defense. Oil contamination is especially harmful to seabirds because oil breaks down the feather barrier, thus impairing waterproofing and provoking water penetration and hypothermia with potential weakening and death of birds (Jenssen, 1994; Piatt & Van Pelt, 1997). In this study, we used visual inspection of the plumage to assess whether eiders were contaminated with oil. However, when using this method, minor traces of oil may have been overlooked and in a few cases confused with blood (Camphuysen, 2005).

Uropygial gland secretion has been proposed to improve water repellency of feathers by providing plumage with a waterproofing layer of lipids (Jacob & Ziswiler, 1982; Møller & Laursen, 2019a), or simply to keep up feather microstructure, which is essential for maintaining the plumage waterproof (Giraudeau et al., 2010; see review in Moreno‐Rueda, 2017). Therefore, a higher release of uropygial gland secretions could minimize the detrimental effect of oil contamination on eiders. The increased Aba (antibacterial activity) with age could be explained as a plastic response of producing larger gland volumes of secretion or secretion with higher Aba against an increasing risk with year; or a selective disappearance of eiders with smaller glands and/or lower Aba from the population (i.e., selection).

Only few studies demonstrate evidence for differences in phenotype between oiled and nonoiled eiders, nor in other seabird species (Champoux et al., 2020). Here, we provide evidence for differences in morphology between these two groups of individuals. We could dismiss the possibility that these differences were due to different origins of eiders since the Baltic‐Wadden Sea population is the only one wintering in the study areas (Laursen & Møller, 2014; Noer, 1991).

Oil pollution has been shown to affect age at maturity, survival rate, and fecundity in extant populations (Alonso‐Alvarez et al., 2007; Votier, Birkhead, et al., 2005; Votier, Hatchwell, et al., 2005; Wolfaardt et al., 2009). There is also some empirical evidence of indirect effects of oil on the marine ecosystem with subsequent effects on eiders and seabirds (Alonso‐Alvarez et al., 2007; Paine et al., 1996; Peterson et al., 2003). We found no evidence of differences in gizzard content and size on probability of oiling as would be expected if the dietary base for eiders had changed because of oil pollution. We found marginal evidence for a change in body condition as a consequence of oil pollution, indicating that if eiders survived the oil spill, the ability to recover is high, which is supported for experiments with gulls (Horak et al., 2020).

The probability of getting oiled was marginally related to age. A previous study of eiders from oil accidents concluded that the age structure was representative for the population (Joensen, 1972). Eiders that had not encountered oil were more likely to be in better body condition and had superior viability than other individuals. We found combined relationships between oiling and a suite of phenotypic traits. The most likely explanation for these results is that morphological characters of eiders reflect their phenotype when contaminated with oil and that the anatomical characters indicate how the bird reacted to oil contamination, thereby facilitating recovery. Large feet, aspect ratio, and wing loading are positively correlated with the probability of eiders being oiled indicating that birds with large locomotor ability are easily contaminated with oil, due to a high activity level, and thus a high probability of encountering oil spills. After contamination, physiological processes are activated to reduce effects as decreased breast muscle mass, which may be reduced to increase metabolic rate to withstand the increased cooling of the body due to oiling of feathers. Breast muscles can be used to store energy and later activate this energy (Laursen et al., 2019). Increased intestine mass may also be part of the restoration process. To maintain metabolic rate and body condition, the birds have to feed indiscriminately, and the large intestine reflects this. This was further supported by large gizzard content, reflecting a high feeding rate, but no change in gizzard mass in oiled birds. The uropygial mass may have increased because secretions from this organ are used to smear the feathers for self‐maintenance and perhaps thereby also clean for oil and toxic substances. Liver mass increased in oiled eiders compared to controls. The liver is the main storage organ for fat‐soluble antioxidants, suggesting that eiders with oil on feathers increase production of antioxidants to reduce the poisonous effects of oil on the body (Møller & Laursen, 2019a). The liver contains antioxidants (Møller et al., 2019) which are useful for maintaining body condition and probably counteract disease due to oil contamination.

Treatment of oiled seabirds has taken place at some coincidence of oil spills (Heubeck et al., 2003; Wolfaardt et al., 2009). The findings reported here may imply that treatment of oiled eiders and other birds should focus on individuals with long wings and large feet because such individuals have a high risk of being oiled, and they would furthermore be more valuable for conservation. However, experiments show that seabirds moderately contaminated with oil recover within some weeks without treatment (Horak et al., 2020).

An alternative interpretation of the results is that it is not the oil and the effects of oil on the environment that affect the birds, but rather effects of morphology that increase the risk of getting oiled. For example, foot area affects diving (Møller & Laursen, 2019b). Wing loading and aspect ratio affect flight ability and maneuverability (Pennycuick, 2008). This implies that body mass loss of oiled birds can reflect loss of condition, or alternatively an adaptive loss of costly locomotion during oiling events (Freed, 1981; Norberg, 1981). Such phenotypic responses to oiling are possible since there were significant relationships between oiling and body condition, gizzard content, wing area, and feather growth.

In conclusion, our study of eiders that survived oil spills showed that eiders with large webbed feet and large morphological characters involved in locomotion in general had higher probability of becoming oiled. In contrast, organs responding to oil contamination by increasing metabolic rate from reduced breast muscle size, increased feeding, and activated organs such as the liver and the uropygial gland may reduce the effects of oiling. These findings imply that oiled eiders do not constitute a random sample, but individuals with superior flight ability as reflected by large wing areas, but also superior diving ability as reflected by large webbed feet are at a selective disadvantage during oil spills. Thus, specific characteristics predispose eiders to oiling, and adaptation to swimming, diving, and flying may be traded against such costs of oiling.

## CONFLICT OF INTEREST

The authors declare no conflicts of interest.

## AUTHOR CONTRIBUTIONS

**Anders Pape Møller:** Conceptualization (equal); Data curation (equal); Formal analysis (lead); Investigation (equal); Methodology (equal); Software (supporting); Supervision (equal); Validation (lead); Visualization (lead); Writing‐original draft (lead); Writing‐review & editing (equal). **Karsten Laursen:** Data curation (lead); Formal analysis (supporting); Funding acquisition (lead); Investigation (supporting); Methodology (supporting); Project administration (lead); Resources (lead); Writing‐original draft (supporting); Writing‐review & editing (equal). **Jorge Izaguirre:** Data curation (supporting); Formal analysis (equal); Investigation (equal); Methodology (equal); Software (supporting); Writing‐original draft (supporting); Writing‐review & editing (equal). **Alfonso Marzal:** Conceptualization (equal); Data curation (supporting); Formal analysis (equal); Investigation (equal); Methodology (equal); Resources (supporting); Software (supporting); Supervision (equal); Validation (equal); Writing‐original draft (supporting); Writing‐review & editing (equal).

## Supporting information

Supplementary Material

## Data Availability

Data are available at https://doi.org/10.5061/dryad.8sf7m0cp3.
