# Supplementary material for: Antibacterial and anatomical defenses in an oil contaminated, vulnerable seaduck
Source: Ecol Evol. 2021 Aug 19;11(18):12520–8. doi: 10.1002/ece3.7996 (PMC8462148; doi:10.1002/ece3.7996)
Supplement: Supplementary file 1 — Supplementary Material [file ECE3-11-12520-s001.docx]

ELECTRONIC SUPPLEMENTARY MATERIAL ESM TABLE 1.

Summary statistics for 27 characters of eiders. The table reports character, lower range, upper range, mean, SE, 95% Upper and Lower Confidence Limits and sample size.

| Character | Lower range | Upper range | Mean | SE | UC | LC | *N* |
| --- | --- | --- | --- | --- | --- | --- | --- |
| Sex | Female | Male |  |  |  |  | 473 |
| Age (years) | 1 | 6 | 5.034 | 0.062 | 5.156 | 4.912 | 473 |
| October date | 1 | 223 | 88.128 | 56.191 | 93.969 | 82.288 | 358 |
| Feather growth bar (mm) | 2.310 | 4.934 | 3.443 | 0.018 | 3.478 | 3.409 | 472 |
| Body mass (g) | 1.79 | 2.71 | 2.273 | 0.013 | 2.298 | 2.247 | 174 |
| Beak volume (cm^3^) | 0.014 | 0.087 | 0.028 | 0.001 | 0.030 | 0.026 | 174 |
| Tarsus length (mm) | 63.591 | 0.277 | 63.591 | 3.628 | 64.137 | 63.046 | 172 |
| Foot area (cm^2^) | 29.557 | 57.508 | 46.059 | 0.404 | 46.857 | 45.261 | 173 |
| Wing length (mm) | 203 | 333 | 303.430 | 0.526 | 304.463 | 302.397 | 472 |
| Wing area (cm^2^) | 463.201 | 630.500 | 545.846 | 1.378 | 548.553 | 543.138 | 460 |
| Wing loading (g/cm^2^) | 0.0031 | 0.0051 | 0.0041 | 0.00003 | 0.0042 | 0.0041 | 173 |
| Feather length (mm) | 184 | 232 | 218.501 | 0.317 | 219.123 | 217.879 | 473 |
| Feather weight (g) | 0.435 | 0.915 | 0.681 | 0.003 | 0.687 | 0.676 | 472 |
| Shaft thickness (mm) | 21.6 | 49.6 | 38.600 | 0.129 | 38.854 | 0.129 | 473 |
| Femur length (mm) | 950.2 | 1222.6 | 1090.064 | 1.880 | 1093.758 | 1086.370 | 463 |
| Feather wear index | 0 | 4 | 0.108 | 0.018 | 0.143 | 0.073 | 472 |
| No. fault bars | 0 | 14 | 0.884 | 0.165 | 1.210 | 0.559 | 199 |
| Duration of molt (days) | 52.076 | 98.27 | 67.551 | 0.500 | 68.536 | 66.566 | 199 |
| Breast muscle mass (g) | 58.825 | 233.597 | 174.855 | 1.890 | 178.586 | 171.123 | 174 |
| Heart mass (g) | 12.663 | 32.238 | 19.772 | 0.295 | 20.355 | 19.189 | 174 |
| Liver mass (g) | 37.375 | 119.020 | 70.340 | 0.991 | 72.297 | 68.383 | 173 |
| Gizzard mass (g) | 108.024 | 3.251 | 108.024 | 3.251 | 114.441 | 101.608 | 174 |
| Gizzard content (g) | 0.008 | 101.367 | 15.182 | 1.166 | 17.484 | 12.880 | 174 |
| Spleen mass (g) | 0.071 | 2.963 | 0.929 | 0.042 | 1.011 | 0.847 | 167 |
| Intestine length (cm) | 77.843 | 236.687 | 151.422 | 2.569 | 156.492 | 146.351 | 174 |
| Uropygial mass (g) | 2.026 | 6.806 | 4.591 | 0.085 | 4.758 | 4.423 | 169 |
| Aspect ratio | 18.569 | 24.229 | 20.923 | 0.072 | 21.065 | 20.780 | 172 |
